# The Arabidopsis bZIP19 and bZIP23 Activity Requires Zinc Deficiency – Insight on Regulation From Complementation Lines

**DOI:** 10.3389/fpls.2018.01955

**Published:** 2019-01-22

**Authors:** Grmay H. Lilay, Pedro Humberto Castro, Ana Campilho, Ana G. L. Assunção

**Affiliations:** ^1^Department of Plant and Environmental Sciences, Copenhagen Plant Science Centre, University of Copenhagen, Frederiksberg, Denmark; ^2^CIBIO/InBIO – Research Centre in Biodiversity and Genetic Resources, University of Porto, Vairão, Portugal; ^3^Department of Biology, University of Porto, Porto, Portugal

**Keywords:** zinc deficiency, *Arabidopsis thaliana*, F-bZIP, regulation, ZIP transporters, plant nutrition

## Abstract

All living organisms require zinc as an essential micronutrient. Maintaining appropriate intracellular zinc supply, and avoiding deficiency or toxic excess, requires a tight regulation of zinc homeostasis. In Arabidopsis, bZIP19 and bZIP23 (basic-leucine zipper) transcription factors are the central regulators of the zinc deficiency response. Their targets include members of the ZIP (Zrt/Irt-like Protein) transporter family, involved in cellular zinc uptake, which are up-regulated at zinc deficiency. However, the mechanisms by which these transcription factors are regulated by cellular zinc status are not yet known. Here, to further our insight, we took advantage of the zinc deficiency hypersensitive phenotype of the *bzip19 bzip23* double mutant, and used it as background to produce complementation lines of each Arabidopsis F-bZIP transcription factor, including bZIP24. On these lines, we performed complementation and localization studies, analyzed the transcript level of a subset of putative target genes, and performed elemental tissue profiling. We find evidence supporting that the zinc-dependent activity of bZIP19 and bZIP23 is modulated by zinc at protein level, in the nucleus, where cellular zinc sufficiency represses their activity and zinc deficiency is required. In addition, we show that these two transcription factors are functionally redundant to a large extent, and that differential tissue-specific expression patterns might, at least partly, explain distinct regulatory activities. Finally, we show that bZIP24 does not play a central role in the Zn deficiency response. Overall, we provide novel information that advances our understanding of the regulatory activity of bZIP19 and bZIP23.

## Introduction

Zinc (Zn) is an essential micronutrient for all living organisms. It is a key structural and catalytic component of a large number of proteins, as cofactor in many enzymes, transcription factors and protein interaction domains. It is estimated that Zn-binding proteins make up to nearly 10% of the proteomes in eukaryotic cells (Coleman, 1992; Andreini et al., 2006). Maintaining appropriate intracellular Zn availability, and avoiding deficiency or toxic excess, requires a tight regulation of Zn uptake, transport, distribution and storage activities (Clemens, 2001). Such regulation of the Zn homeostasis network is finely tuned according to Zn intracellular levels, fluxes, and external fluctuations. One of the primary means by which cells can regulate their Zn levels is through Zn-dependent changes in the expression of genes required for Zn transport and storage (Choi and Bird, 2014). Members of the ZIP (Zrt/Irt-like Protein) and CDF (Cation Diffusion Facilitator) transporter families are ubiquitously used in eukaryotes for Zn uptake and efflux, into and out of the cytosol, respectively (Eide, 2006; Blindauer, 2015). Understanding how changes in cellular Zn status are sensed and affect the expression of Zn homeostasis genes is important, in particular to know how organisms respond to Zn deficiency.

Most knowledge on how eukaryotic cells sense Zn deficiency comes from yeast model systems. In *Saccharomyces cerevisiae* the Zn-responsive transcriptional activator Zap1 plays a central role in maintaining Zn homeostasis (Zhao et al., 1998). In Zn-limited cells Zap1 binds to *Zinc Responsive Elements (ZRE)* in target gene promoters activating gene expression. These include plasma membrane and tonoplast localized ZIP family members, among others, involved in increased Zn uptake transport to the cytosol. This regulatory activity involves independent mechanisms in which Zn binds activation domains (AD1, AD2) and the DNA binding domain of Zap1, resulting in either sensing of cellular Zn deficiency or limiting Zap1 activity in the presence of Zn (Zhao et al., 1998; Lyons et al., 2000; Bird et al., 2003; Frey et al., 2011).

In *Arabidopsis thaliana* (Arabidopsis), bZIP19 and bZIP23, two members of the basic-leucine zipper (bZIP) dimerizing transcription factor family, are involved in the regulation of Zn homeostasis, being the central regulators of the Zn deficiency response (Assunção et al., 2010; Inaba et al., 2015). They belong to the three-member F subfamily of Arabidopsis bZIP proteins (F-bZIP), which also includes bZIP24, known to be involved in salt stress tolerance regulation (Jakoby et al., 2002; Popova et al., 2008; Yang et al., 2009). The double mutant *bzip19 bzip23* (*bzip19/23*) is hypersensitive to Zn deficiency and has no apparent phenotype on control growth conditions. The bZIP19 and bZIP23 bind to a 10 bp *Zinc Deficiency Response Element* (*ZDRE*, RTGTCGACAY) present in the promoter region of their putative target genes (Assunção et al., 2010) which include members of the Arabidopsis ZIP transporter family (i.e., *ZIP1/3/4/5/9/10/12 and IRT3*). Expression of these *ZIP* genes is up-regulated in response to Zn deficiency and is unresponsive in the *bzip19/23* mutant background (Assunção et al., 2010). However, the specific physiological roles of ZIP transporters in plant Zn homeostasis is still incompletely understood (Sinclair and Krämer, 2012).

Among the *ZIP* putative target genes of bZIP19 and bZIP23, yeast complementation studies showed that ZIP1/3/4/12 and IRT3 mediate Zn uptake (Grotz et al., 1998; Lin et al., 2009; Assunção et al., 2010; Milner et al., 2013), and IRT3 and ZIP1 localize to the plasma membrane and vacuolar membrane, respectively (Lin et al., 2009; Milner et al., 2013). It is however *ZIP4* that offered most insight on its role in the bZIP19/23 regulatory network. Both transcription factors bind to promoter fragments of *ZIP4*, containing two *ZDRE* motifs (Assunção et al., 2010), where the most upstream one is necessary for gene expression (Lin et al., 2016). Moreover, *in vivo ZIP4* promoter GUS reporter assay, in wild-type and *bzip19/23* mutant backgrounds, indicated that the bZIP19 and bZIP23 transcription factors act as the main regulators of *ZIP4* (Castro et al., 2017). Together with a strong root and shoot GUS reporter expression of *ZIP4* in response to Zn deficiency (Lin et al., 2016), the evidence points at an important role of ZIP4 transporter in the Zn deficiency response. The small set of bZIP19/23 putative target genes also include nicotianamine synthase (NAS) genes, *NAS2* and *NAS4*, whose transcription is induced by Zn deficiency (Weber et al., 2004; van de Mortel et al., 2006; Assunção et al., 2010). Nicotianamine (NA) is produced by NAS enzyme and it is a low-molecular-weight metal chelator suggested to play an important role in iron (Fe) and Zn homeostasis, involved in their distribution and movement within the plant (Curie et al., 2009; Deinlein et al., 2012; Schuler et al., 2012; Clemens et al., 2013).

While gaining more knowledge on the target genes of bZIP19 and bZIP23, the mechanisms by which these transcription factors are regulated by cellular Zn status are still unknown (Assunção et al., 2013). Although there is a modest increase of *bZIP19* and *bZIP23* transcript levels at Zn deficiency (Assunção et al., 2010; Inaba et al., 2015), Zn might directly or indirectly affect protein function and regulate the transcription factors activity. This could involve, for example, protein subcellular localization, dimerization properties, DNA binding activity or protein-protein interactions (Choi and Bird, 2014). Here, to further our insight on the effect of cellular Zn status on the bZIP19 and bZIP23 activity, we took advantage of the Zn deficiency hypersensitive phenotype of the *bzip19/23* double mutant, and used it as background to obtain stably transformed lines constitutively overexpressing each of the F-group *bZIP* genes. On these lines, we performed complementation and localization studies, analyzed the transcript level of a subset of putative target genes (*ZIP4*, and also *ZIP1, ZIP5, NAS*2 and *NAS4*), and performed elemental profiling of plant tissue. Our analyses also included complementation with bZIP24, the third member of the F-bZIPs, to examine any functional relation to the Zn deficiency response. The results provided novel information that advances our understanding of the Zn sensing and regulatory activity of bZIP19 and bZIP23.

## Materials and Methods

### Plasmid Construction and Plant Transformation

The *pCaMV35S::bZIP-CFP-HA* constructs for stable transformation of the Arabidopsis *bzip19/23* mutant were generated as follows: the full-length CDS of *bZIP19* (At4g35040), *bZIP23* (At2g16770), and *bZIP24* (At3g51960) were amplified from an Arabidopsis cDNA library using forward and reverse primers containing *Not*I and *Asc*I restriction sites, respectively (Supplementary Table S1). The PCR products were purified by kit (PureLink, Invitrogen) and cloned into pGEM-T Easy vector using T4 DNA Ligase (Promega), then cloned into the *Not*I/*Asc*I restriction enzyme sites of the pENTR^TM^/D-TOPO vector (Invitrogen), followed by *in vitro* site-directed recombination into pEarleyGate 102 Gateway vector (Earley et al., 2006), carrying a *CaMV35S* promoter, a C-terminal CFP fluorophore and HA-tag, using LR Clonase^TM^ II Enzyme Mix (Invitrogen). All constructs were verified by restriction enzyme digestion analysis and sequencing. The constructs were transformed into *Agrobacterium tumefaciens* strain GV3101 (pMP90) by electroporation and used for Arabidopsis *bzip19/23* mutant transformation by the Floral dip method (Clough and Bent, 1998). Transgenic plants were selected by Basta (phosphinothricin) resistance and homozygous transgenic seeds (T3 generation) of three to four independent lines per construct, were selected. The overexpression of each *F-bZIP* gene was confirmed in the respective lines by real-time quantitative RT-PCR (RT-qPCR). The lines were referred to as *bzip19/23*-OE19, *bzip19/23*-OE23 and *bzip19/23*-OE24. The *pCaMV35S::GFP-bZIP* constructs for transient transformation of *Nicotiana benthamiana* leaves were generated as described above by *in vitro* site-directed recombination from the pENTR^TM^/D-TOPO constructs into pMCD43 Gateway vector (Curtis, 2003), carrying a *CaMV35S* promoter and a N-terminal GFP fluorophore, using LR Clonase^TM^ II Enzyme Mix (Invitrogen). Transformed *A. tumefaciens* clones, obtained as previously described and containing each construct confirmed by PCR, were grown overnight at 28°C in LB media supplemented with selective antibiotics. Cell cultures were centrifuged 15 min at 3000 *g* and the pellet suspended in MgCl_2_ 10 mM solution to reach an OD_600_ of 0.5. Bacteria solutions were incubated with gentle shaking at room temperature for 2–3 h prior to syringe-inoculation into the leaves of 4–5 week old *N. benthamiana* plants at the abaxial side. In order to generate promoter-GUS fusion lines, the promoter regions (the whole intergenic region upstream the start codon) of *bZIP19* and *bZIP23* were amplified from Arabidopsis genomic DNA and cloned into the *Not*I/*Asc*I restriction enzyme sites of the pENTR^TM^/D-TOPO vector as described above (Supplementary Table S1), followed by *in vitro* site-directed recombination into pBGWFS7 Gateway vector (Karimi et al., 2002) carrying a C-terminal GFP fluorophore and GUS reporter gene, using LR Clonase^TM^ II Enzyme Mix (Invitrogen). Transformation of *A. tumefaciens* and Arabidopsis were performed as previously described. Transgenic plants were selected by Basta resistance and seeds (T2 generation) of 10 independent lines per construct, were analyzed. They were referred to as *pbZIP19::GUS* and *pbZIP23::GUS* lines.

### Plant Material and Growth Conditions

Arabidopsis wild-type (accession Columbia, Col-0) and the *bzip19/23* double mutant (Col-0 background, descibed in Assunção et al., 2010) were used in all experiments. Synchronized seeds were stratified in water at 4°C for 3 days and surfaced sterilized by soaking for 5 min in 1 mL 70% (v/v) ethanol, followed by 10 min incubation in 1 mL of bleach solution [20% (v/v) commercial bleach with 3.5% (w/v) effective chloride; 0.1% (v/v) Tween-20]. Seeds were rinsed 5 times with sterile ultrapure water (Milli-Q Element, Merck) and resuspended in a sterile 0.25% (w/v) agarose solution. For the analysis with MS grown seedlings, sterilized seeds were sown on half-strength murashige and skoog (MS) medium with 1.5% (w/v) sucrose, 0.5 g L^-1^ MES and 1.2% (w/v) phyto-agar (Duchefa) at pH 5.7 in 120 mm square plastic Petri dishes, with 50 mL medium per plate, placed vertically. The glassware used for the preparation of the MS medium was previously rinsed with 0.1N HCl solution followed by 5 times rinsing with ultrapure water. The medium was prepared with ultrapure water and contained 15 μM ZnSO_4_ (control; Zn sufficient media) or no added Zn (-Zn; Zn-deficient media). The wild-type, *bzip19/23* mutant, *bzip19/23*-OE19, *bzip19/23*-OE23, and *bzip19/23*-OE24 lines were grown together in plates (*ca.* 5 seedlings per genotype) at control or –Zn MS media, for 10 or 14 days. For each transformed line, three independent lines were tested, with at least four plates (replicates) per independent line and per Zn condition. For the analysis with hydroponically grown plants, sterilized seeds of the above mentioned lines were germinated on 1.5 mL plastic microtubes, cut open *ca.* 2 cm from the bottom and filled with 0.8% (w/v) phyto-agar solution. The microtubes were placed inside a hole at the lead of 50 mL plastic tubes cut open at the bottom, and these were placed in the lead of a 10 L container, which held 8 × 6 holes. A modified half-strength Hoagland nutrient solution was used, containing: 2 mM Ca(NO_3_)_2_, 1 mM MgSO_4_.7H_2_O, 3 mM KNO_3_, 1 mM KH_2_PO_4_, 25 μM Fe-Na-EDTA, 25 μM H_3_BO_3_, 3 μM MnSO_4_.H_2_O, 0.1 μM CuSO_4_.5H_2_O, 0.5 μM (NH_4_)_6_Mo_7_O_24_, 50 μM KCl, with 1 mM MES at pH 5.7, with either 2 μM ZnSO_4_.7H_2_O (control; Zn sufficient media) or with 0.002 μM ZnSO_4_.7H_2_O (-Zn; Zn-deficient media). The plastic containers, for the control and –Zn treatments, were rinsed with 0.1 N HCl solution followed by 5 times rinsing with ultrapure water prior to use, and the nutrient solutions were prepared with ultrapure water. Seeds were germinated and grown for 8 weeks in either control or -Zn nutrient solution, with 6 plants per genotype, in a 10 L container, which was aerated throughout the experiment. The nutrient solutions were replaced once a week, during the first 4 weeks, and twice in the weeks thereafter. The hydroponics setup and the MS plates were in a growth chamber with 8/16 h and 16/8 h light/dark cycle, respectively, 125 μmol m^-2^ s^-1^ white light, 22/20°C light/dark temperature, and 70% relative humidity.

### Subcellular Localization Analysis

Ten-day-old seedlings of wild-type, *bzip19/23* mutant, *bzip19/23*-OE19, *bzip19/23*-OE23 and *bzip19/23*-OE24 lines were grown in control or –Zn MS medium. For laser scanning confocal microscopy (LSCM) analysis, roots were transferred to microscope slides containing propidium iodide (PI) to stain root cell walls and were visualized using a laser scanning confocal inverted microscope Leica TCS SP5 II (Leica Microsystems) with a HC PL APO CS 63x /1.30 Glycerine objective. Ar 458 nm and 514 nm laser lines were used for CFP and PI excitation, respectively. The emission settings were between 470–500 nm for CFP and 590–630 nm for PI. For each transformed line, three independent lines were tested, with observations of *ca.* 2–3 seedlings per independent line and Zn condition. For the analysis with *N. benthamiana*, three plants were infiltrated per construct and were visualized 4–5 days after infiltration using a laser scanning confocal microscope Leica TCS SP5-X (Leica Microsystems) with a HCX PL APO 20x /0.70 water objective. White light laser at 480 nm was used for GFP excitation. The emission settings were between 500–580 nm for GFP, 631–721 nm for far-red with transmission (bright filed) also recorded.

### Real-Time Quantitative RT-PCR Analysis

Fourteen-day-old seedlings of wild-type, *bzip19/23* mutant, *bzip19/23*-OE19, *bzip19/23*-OE23 and *bzip19/23*-OE24 lines, grown in control or –Zn MS medium were harvested and immediately frozen in liquid nitrogen in pools of 5 seedlings per line and per Zn treatment × 3 different plates grown simultaneously and considered as biological replica. Seedlings were grinded in a microtube in liquid nitrogen, with the help of polypropylene pestles. Total RNA was extracted using the RNeasy Plant Mini Kit (QIAGEN), and RNA quantity and integrity were assessed using both a Nanodrop spectrophotometer (Thermo Scientific), and standard agarose-gel electrophoretic analysis. The RNA samples were treated with Recombinant DNase I (Sigma-Aldrich) and first strand cDNA synthesis was generated from 0.5 μg total RNA using a SuperScript^TM^ III First-Strand Synthesis SuperMix (Invitrogen). Primers for RT-qPCR (Supplementary Table S2) were designed using NCBI Primer-BLAST^1^ and the primer amplification efficiency for each primer pair was between 1.9 and 2.1. RT-qPCR was performed with a LightCycler 96 Real-Time PCR Systtem (Roche Diagnostics) in 96-well plates, using HOT FIREPol EvaGreen qPCR Mix (Solis BioDyne) in the PCR reaction mixture, according to manufacturer’s instructions (i.e., final volume of 20 μL containing 4 μL of the EvaGreen qPCR mix, 1 μL of 10 μM primers, and 1 μL of a 1/10 dilution of cDNA as template). The PCR conditions were as follows: 95^o^C for 15 min, followed by 40 cycles of 95^o^C for 15 s, 60^o^C for 20 s, and 72^o^C for 20 s, with a final step at 98^o^C. *ACT2* (At3g18780) was used as the reference gene. Reactions were performed in 2–3 technical replicas per biological replica and in 3 biological replica per line and per Zn treatment. The calculated cycle threshold (Ct) value for each gene was normalized to the control *ACT2* gene calculated Ct value. The relative transcript levels were expressed against the wild-type seedlings grown at control conditions, and calculated according to the Livak 2^-ΔΔCT^ method (Livak and Schmittgen, 2001).

### Tissue Elemental Analysis by ICP-MS

Shoot and root fresh tissue was harvested from 8-week-old hydroponically grown plants from wild-type, *bzip19/23* mutant, *bzip19/23*-OE19, *bzip19/23*-OE23 and *bzip19/23*-OE24 lines, grown at control or –Zn nutrient solution. Roots were desorbed with ice-cold 1 mM CaCl_2_ solution for 15 min followed by 3 times washing with ultrapure water for 1 min. Shoot and root tissue from 4 plants per line were harvested separately, blotted dry, dried for 3 days at 60°C and the shoot and root total dry weight measured. For *bzip19/23* mutant and *bzip19/23*-OE24 lines grown at –Zn, 6 plants were harvested, following the same procedure, and were analyzed in pools of 2. Tissue digestion was performed with ultra-pure acids (70% HNO_3_ and 15% H_2_O_2_) at 240°C and 80 bars for 15 min in a pressurized microwave oven (Ultrawave, Milestone Inc.) enabling the amount of acid and final dilution to be adjusted according to the amount of plant material (Olsen et al., 2016). All samples were diluted to 3.5% HNO_3_ prior to multi-elemental analysis using Inductively-Coupled Plasma Mass Spectrometry (ICP-MS) (7900 ICP-MS, Agilent Technologies) run in collision mode (Helium). The ICP-MS was equipped with a SeaSpray nebuliser and a double pass scott type spray chamber, moreover, automatic sample introduction was performed from an 89 position Integrated autosampler (Agilent Technologies). Certified reference material (NIST1515, apple leaf, National Institute of Standards and Technology, United States) was included to evaluate data quality and drift correction was performed based on drift samples for every 10 samples. Data were processed using Agilent ICP Masshunter Software version 4.3.

### Histochemical Staining for β-Glucuronidase (GUS) Assay

Histochemical GUS staining was performed in 14-day-old seedlings of *pbZIP19::GUS* and *pbZIP23::GUS* lines grown in control or –Zn MS medium. Seedlings were immersed in GUS staining solution containing 50 mM phosphate buffer, 10 mM Na_2_-EDTA, 20% (v/v) methanol, 0.1% (v/v) Triton X-100, 1.4 mM K_3_[Fe(CN)_6_], 1.4 mM K_4_[Fe(CN)_6_].3H_2_O with 1.9 mM X-Gluc and incubated overnight at 37^o^C in the dark (Jefferson et al., 1987). After incubation, the pigments were removed by repeated incubations in 50, 70, and 96% (v/v) ethanol successively, and seedlings were stored in 70% (v/v) glycerol.

### Statistical Analysis

One-way ANOVA followed by Tukey’s *post hoc* test, and Student’s *t*-test were calculated with IBM SPSS Statistics V22.0 software.

## Results

### Complementation Study in *bzip19/23* Double Mutant Background

In order to obtain complementation lines of the Arabidopsis F-bZIPs (bZIP19, bZIP23 and bZIP24) in the *bzip19/23* double mutant background, we produced stably transformed lines expressing each of the *F-bZIPs* cDNA under control of the constitutive *CaMV 35S* promoter and containing a C-terminal CFP fluorophore (i.e., *bzip19/23-*OE19, *bzip19/23-*OE23 and *bzip19/23-*OE24). We found an increased transcript level of the overexpressed genes in these lines (Supplementary Figure S1), and a western blot (WB) anti-HA, albeit only tested in the *bzip19/23*-OE19 line, confirmed the expected protein molecular weight for the bZIP19-CFP-HA expressed protein (Supplementary Figure S2 and Supplementary Methods). The latter also indicated a similar protein amount at control and Zn-deficient growth conditions.

**FIGURE 1:**
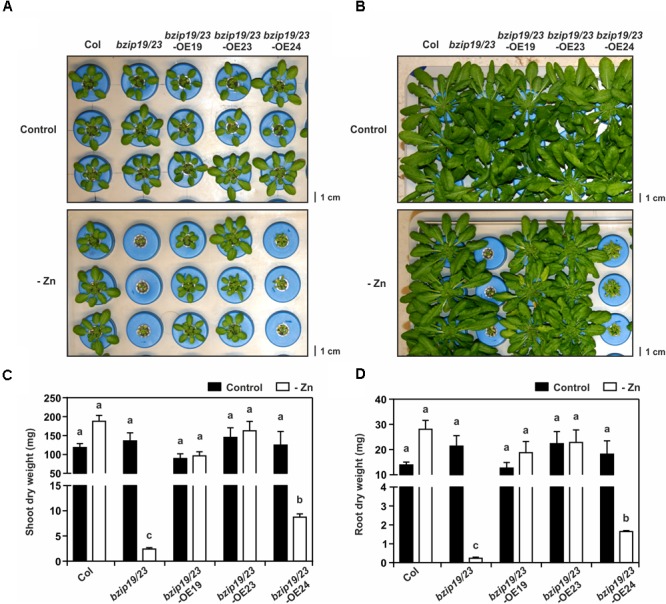
Complementation study with Arabidopsis *bzip19/23*-OE19, *bzip19/23*-OE23 and *bzip19/23*-OE24 lines, and wild-type (Col) and *bzip19/23* double mutant lines, grown in hydroponics with control (control) or Zn-deficient (-Zn) nutrient solution. Phenotype of 4-week-old plants **(A)** and 8-week-old plants **(B)**, Shoot **(C)**, and root **(D)** dry weight (mg) of 8-week-old plants grown at control (black bar) or –Zn (open bar). Data are represented as means ± SE (*n* = 4 for all treatments, except *bzip19/23* and *bzip19/23*-OE24 at –Zn with *n* = 3). Different letters indicate significant differences (*p* < 0.05) after one-way ANOVA followed by Tukey’s *post hoc* test.

To test the complementation of the *bzip19/23* mutant Zn deficiency hypersensitive phenotype, the lines were grown in hydroponics under control or Zn-deficient conditions. The *bzip19/23*-OE19 and *bzip19/23*-OE23 lines complemented the *bzip19/23* phenotype at Zn deficiency, displaying normal growth, comparable to the wild-type. The *bzip19/23*-OE24 line, on the other hand, showed severe growth impairment under Zn deficiency, comparable with the *bzip19/23* mutant (Figure 1A). The plants were allowed to grow 8 weeks before harvesting plant material for tissue elemental analyses. Throughout the experiment, the same phenotype pattern was maintained, with *bzip19/23* and *bzip19/23*-OE24 plants displaying an even more severe Zn deficiency hypersensitive phenotype. The rosette of *bzip19/23*-OE24 plants appeared however slightly bigger than the ones from the *bzip19/23* mutant (Figure 1B). The tissue dry weight data were in agreement with these observations (Figures 1C,D). We also analyzed the lines at control or Zn-deficient MS medium, with *bzip19/23*-OE19 and *bzip19/23*-OE23 seedlings complementing the *bzip19/23* mutant, whereas the 5-day-old and 14-day-old *bzip19/23*-OE24 seedlings, at Zn deficiency, displayed shorter root length than the wild-type, but did not show the Zn deficiency phenotype of *bzip19/23* mutant (Supplementary Figures S3A,B).

### Expression and Subcellular Localization Analysis Under Different Zn Supply

**FIGURE 2:**
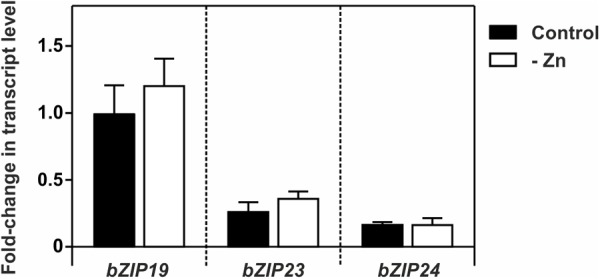
Transcript level profiles of Arabidopsis *bZIP19, bZIP23* and *bZIP24* using real-time quantitative RT-PCR, in 14-day-old wild-type seedlings grown in control (control, dark bars) or Zn-deficient (-Zn, open bars) MS medium. Bars represent mean fold-change in transcript level of three biological replica ± SE. There were no statistically significant differences between control and –Zn, determined by Student *t*-test.

We analyzed the expression of *bZIP19, bZIP23*, and *bZIP24* in wild-type seedlings grown at control or Zn deficiency. Their transcript levels were in line with previous data (Assunção et al., 2010), and indicated a small but not significant increase in *bZIP19* and *bZIP23* transcript abundance at Zn deficiency, not visible in *bZIP24* (Figure 2).

**FIGURE 3:**
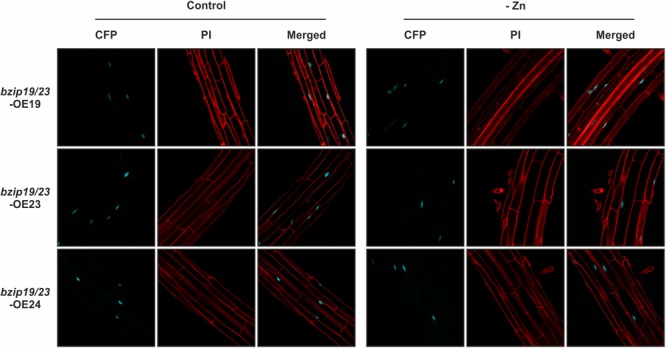
Subcellular localization analysis of bZIP19-CFP, bZIP23-CFP, and bZIP24-CFP fusion proteins in roots of 10-day-old seedlings of Arabidopsis *bzip19/23*-OE19, *bzip19/23*-OE23, and *bzip19/23*-OE24 lines, respectively, grown in control (control) or Zn-deficient (-Zn) MS medium. Emissions of CFP and propidium iodide (PI) were visualized with confocal laser scanning microscopy (CLSM).

We further investigated the subcellular localization of the Arabidopsis F-bZIP proteins, and whether it is affected by cellular Zn status, in seedlings of *bzip19/23*-OE19, *bzip19/23*-OE23 and *bzip19/23*-OE24 lines, grown at control or Zn-deficiency (Supplementary Figure S3). The fluorescence of the C-terminal CFP fluorophore was visualized with confocal laser scanning microscopy (CLSM). The fluorescent signal for the three F-bZIP protein fusions (bZIP19-CFP, bZIP23-CFP, and bZIP24-CFP) localized at the cell nucleus. Our analysis also revealed that the subcellular localization pattern observed for all F-bZIPs was identical between seedlings grown at control or Zn-deficiency (Figure 3). In addition, we performed a localization analysis using transient expression in *N. benthamiana* leaves with each Arabidopsis *F-bZIP* cDNA under control of the constitutive *CaMV 35S* promoter and containing an N-terminal GFP fluorophore. We again found a nuclear localization for the three F-bZIP proteins, thus showing agreement between the results with a C- and N-terminal fluorophore (Supplementary Figure S4).

### Expression Analysis of bZIP19/23 Putative Target Genes

**FIGURE 4:**
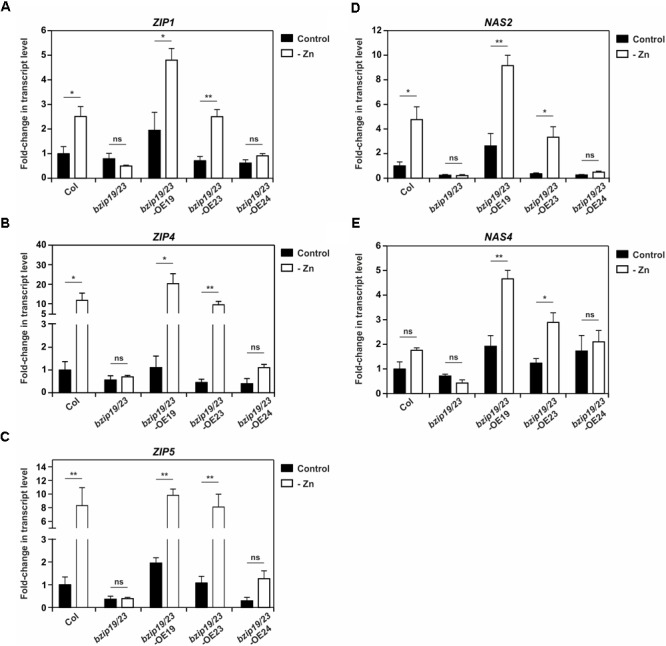
Transcript level profiles of Arabidopsis bZIP19 and bZIP23 putative target genes in 14-day-old seedlings of wild-type (Col), *bzip19/23* double mutant, *bzip19/23*-OE19, *bzip19/23*-OE23 and *bzip19/23*-OE24 lines, grown in control (control, dark bars) or Zn-deficient (-Zn, open bars) MS medium. Real-time quantitative RT-PCR was used to determine the transcript levels of *ZIP1* **(A)**, *ZIP4* **(B)**, *ZIP5* **(C)**, *NAS2* **(D)**, and *NAS4* **(E)**. Bars represent mean fold-change in transcript level of three biological replica ± SE. Statistically significant differences between control and –Zn were determined by Student *t*-test (^∗^*p* < 0.05; ^∗∗^*p* < 0.01; ns, not significant).

Next, we investigated the expression of a subset of the bZIP19 and bZIP23 putative target genes (*ZIP1, ZIP4, ZIP5, NAS*2, and *NAS4*), in seedlings of *bzip19/23*-OE19, *bzip19/23*-OE23 and *bzip19/23*-OE24 lines, grown at control or Zn-deficiency. The analysis of *ZIP1, ZIP4*, and *ZIP5* showed induction of gene expression at Zn deficiency in the wild-type, in particular for *ZIP4* and *ZIP5*, whereas their transcript levels in the *bzip19/23* mutant, in both Zn conditions, were comparable or lower to the wild-type at control. In *bzip19/23*-OE19 and *bzip19/23*-OE23 lines their expression profiles were overall comparable with the wild-type, though with higher transcript level for *ZIP1* and *ZIP4* in *bzip19/23*-OE19. The *bzip19/23*-OE24 line, on the other hand, exhibited transcript levels comparable with the *bzip19/23* mutant, but showing a small induction of *ZIP4* and *ZIP5* at Zn deficiency (Figures 4A–C). The analysis of *NAS2* and *NAS4* showed expression profiles in the different lines comparable to that of the *ZIP* genes. The wild-type showed Zn deficiency induction, though not significant for *NAS4*, whereas *bzip19/23* had transcript levels comparable or lower to the wild-type at control. The expression of *NAS2* and *NAS4* in *bzip19/23*-OE19 and *bzip19/23*-OE23 lines were comparable with the wild-type, but with a pronounced Zn deficiency induction of *NAS4* and with higher transcript levels in *bzip19/23*-OE19. In the *bzip19/23*-OE24 line the transcript levels of *NAS2* were comparable with the *bzip19/23* mutant, whereas *NAS4* exhibited some increased expression at both Zn conditions (Figures 4D,E).

### Tissue Elemental Analysis of Hydroponically Grown Plants

**FIGURE 5:**
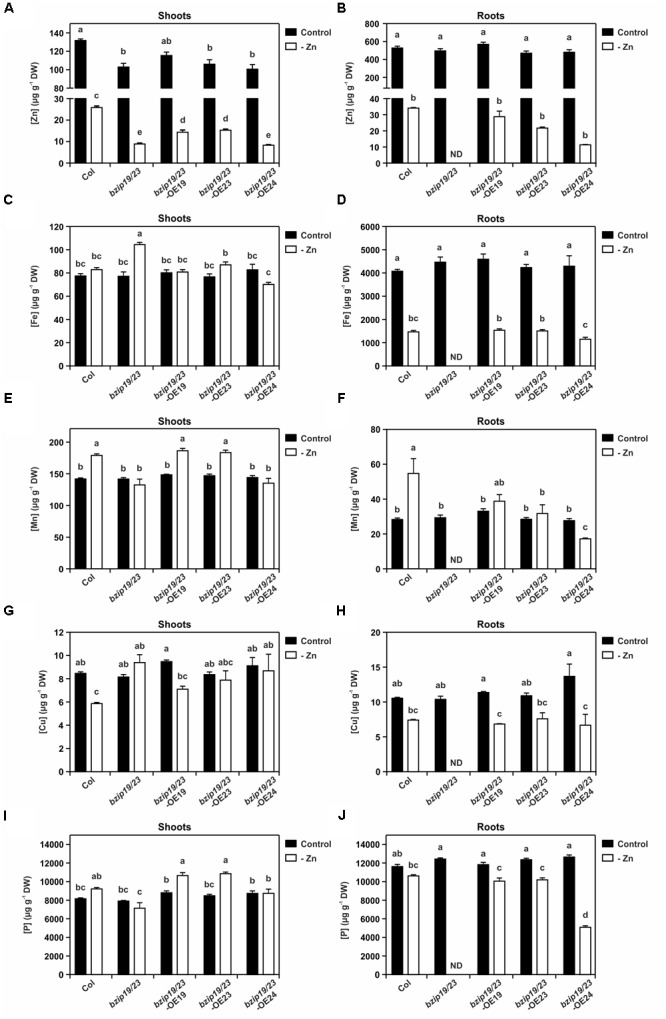
Tissue elemental profiling determined with inductively coupled plasma-mass spectrometry (ICP-MS) in 8-week-old plants of Arabidopsis wild-type (Col), *bzip19/23* double mutant, *bzip19/23*-OE19, *bzip19/23*-OE23 and *bzip19/23*-OE24 lines, grown in hydroponics with control (control, dark bars) or Zn-deficient (-Zn, open bars) nutrient solution. Zn concentration in shoots **(A)**, and in roots **(B)**, Fe concentration in shoots **(C)**, and in roots **(D)**, Mn concentration in shoots **(E)**, and in roots **(F)**, Cu concentration in shoots **(G)**, and in roots **(H)**, P concentration in shoots **(I)**, and in roots **(J)**, in μg g^-1^ dry weight (DW). Data are represented as means ±*SE* (sample sizes as in Figure 1, except for *bzip19/23* roots at –Zn, with no data, ND). Different letters indicate significant differences (*p* < 0.05) after one-way ANOVA followed by Tukey’s *post hoc* test.

To further investigate the complementation of the *bzip19/23* mutant with the Arabidopsis *F-bZIPs*, we analyzed the elemental profiles of shoot and root tissue from the *bzip19/23*-OE19, *bzip19/23*-OE23, and *bzip19/23*-OE24 lines grown in hydroponics at control or Zn-deficiency. The root tissue harvested from the *bzip19/23* mutant plants was extremely small, in line with the severity of the Zn deficiency phenotype (Figure 1), not being adequate to include in the analysis. The results from the elemental profiling revealed that, at control conditions, the element concentrations in shoots and roots did not differ strongly between wild-type, mutant and complementation lines (Figure 5 and Supplementary Table S3).

At Zn deficiency however, there were significant differences in tissue element concentrations between the lines. The Zn content in shoots of *bzip19/23* mutant and *bzip19/23*-OE24 lines was significantly lower than in the wild-type and the *bzip19/23*-OE19 and *bzip19/23*-OE23 lines, with these having lower content than the wild-type (Figure 5A). Overall, shoot and root Zn content in all lines at Zn deficiency decreased to *ca.* 0.1 that of the control (Figures 5A,B). With respect to other analyzed elements, the Fe content in shoots at Zn-deficient conditions was similar to control conditions, except for the *bzip19/23* mutant and *bzip19/23*-OE24, which were significantly higher and lower, respectively (Figure 5C). The Fe content in roots of wild-type and complementation lines, on the other hand, decreased at Zn deficiency to *ca.* 0.3 that of the control (Figure 5D). The manganese (Mn) content in shoots of wild-type, *bzip19/23*-OE19 and *bzip19/23*-OE23 lines at Zn deficiency was significantly higher than *bzip19/23* mutant and *bzip19/23*-OE24 lines. These exhibited similar content in both Zn conditions, whereas wild-type, *bzip19/23*-OE19 and *bzip19/23*-OE23 lines increased to *ca.* 1.3 that of the control (Figure 5E). Also in roots, the Mn content at Zn deficiency increased in wild-type, in *bzip19/23*-OE19 and, to a lesser extent, in *bzip19/23*-OE23, and decreased in *bzip19/23*-OE24 in relation to control (Figure 5F). The content of copper (Cu) in roots of wild-type and complementation lines at Zn deficiency decreased to *ca.* 0.6 that of the control, while in shoots it decreased in wild-type and *bzip19/23*-OE19 lines (Figures 5G,H). The phosphorus (P) content in shoots of wild-type, *bzip19/23*-OE19 and *bzip19/23*-OE23 lines at Zn deficiency increased to *ca.* 1.2 that of the control, whereas *bzip19/23* mutant and *bzip19/23*-OE24 remained comparable at both Zn conditions (Figure 5I). In roots, the wild-type and complementation lines showed a decrease in P content from control to Zn deficiency, which was more pronounced in *bzip19/23*-OE24 than in the wild-type, *bzip19/23*-OE19 and *bzip19/23*-OE23 lines (Figure 5J).

### Tissue Expression Analysis of *bZIP19* and *bZIP23*

**FIGURE 6:**
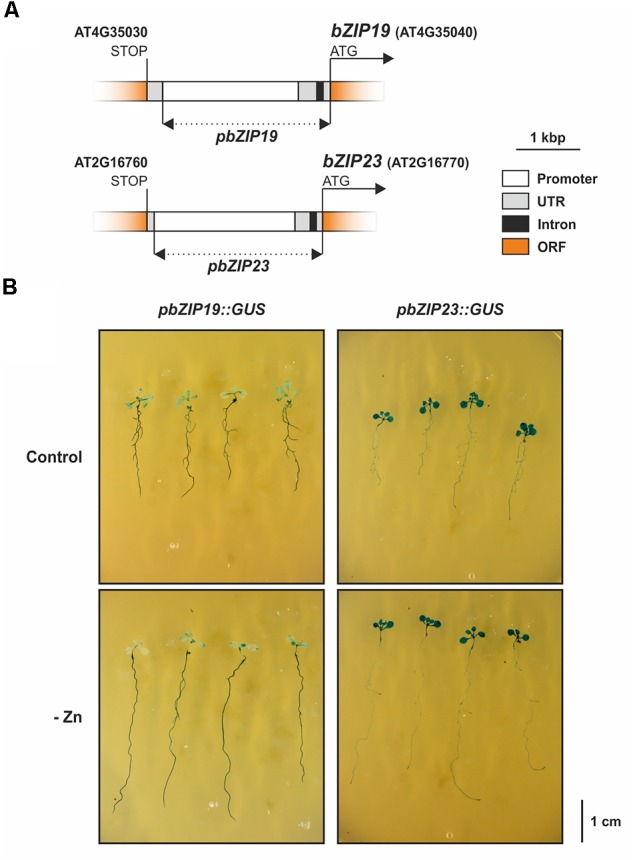
Arabidopsis *bZIP19* and *bZIP23* tissue expression analysis with promoter-reporter gene fusions. Schematic representation of the *bZIP19* and *bZIP23* promoter regions, represented by a dashed line, cloned for promoter::GUS fusion constructs **(A)**, histochemical staining for β-glucuronidase (GUS) assay in 14-day-old seedlings of *pbZIP19::GUS* and *pbZIP23::GUS* lines, grown in control (control) or Zn-deficient (-Zn) MS medium **(B)**.

Lastly, we studied tissue-specific expression of *bZIP19* and *bZIP23* by producing stably transformed lines in wild-type Arabidopsis background with the *bZIP19* or *bZIP23* promoters fused to β-glucuronidase (GUS), *pbZIP19::GUS* and *pbZIP23::GUS* lines (Figure 6A). The histochemical GUS staining assay of seedlings grown at control or Zn-deficiency revealed differences between the two lines. The *pbZIP19::GUS* seedlings showed a stronger reporter GUS expression in roots than in shoots, whereas the *pbZIP23::GUS* seedlings showed the opposite pattern, with a stronger expression in shoots than in roots (Figure 6B). In addition, there were no differences in the reporter GUS expression between seedlings grown at control or Zn-deficiency for both lines.

## Discussion

### The Activity of bZIP19 and bZIP23 Transcription Factors Requires Zn Deficiency Sensing

We found a modest effect of Zn supply on the *bZIP19* and *bZIP23* transcript levels, and no noticeable effect for *bZIP24*, in line with previous report (Assunção et al., 2010). In addition, our promoter-reporter fusion analysis of *bZIP19* and *bZIP23* showed no visible differences between seedlings grown at control or Zn-deficient conditions. This suggests that the Zn-dependent activity of bZIP19 and bZIP23 is not modulated by Zn at the transcriptional level. At the protein level, our results showed a nuclear localization of bZIP19 and bZIP23 in roots of the *bzip19/23-*OE19 and *bzip19/23*-OE23 lines. This is in line with previously reported nuclear localization of bZIP19 and bZIP23 in roots of Arabidopsis bZIP19-GFP and bZIP23-GFP overexpressor lines (Inaba et al., 2015). We further showed that the protein nuclear localization is unaffected when seedlings are grown at control or Zn-deficiency. This strongly indicates that changes in cellular Zn level do not interfere with protein subcellular localization, and likely do not interfere with protein stability. Thus a Zn-dependent change in protein subcellular localization does not seem to be a mechanism by which Zn regulates the activity of bZIP19 and bZIP23 transcription factors.

The expression profiles of a subset of putative target genes of bZIP19/23 provided novel insight on how cellular Zn status impacts on bZIP19 and bZIP23 activity. As previously known, the *bzip19/23* mutant fails to induce the expression of *ZIP1, ZIP4, ZIP5, NAS2*, and *NAS4* at Zn deficiency (Assunção et al., 2010; Castro et al., 2017). Our transcript level analyses showed that the wild-type expression patterns were generally restored in the *bzip19/23-*OE19 and *bzip19/23*-OE23 lines, and also, their tissue elemental analysis showed a largely similar pattern to the wild-type. This is consistent with the complementation of the *bzip19/23* mutant in these lines, supporting the role of bZIP19 and bZIP23 as central players in the Zn deficiency response. Interestingly, our results showed target gene expression induction at Zn-deficiency in *bzip19/23-*OE19 and *bzip19/23*-OE23 lines, likewise in the wild-type, suggesting that the activity of bZIP19 and bZIP23 transcription factors requires Zn deficiency and is repressed by cellular Zn sufficiency. Both transcription factors contain a region that is rich in cysteine and histidine residues located at their N-terminus which was hypothesized to be a Zn-binding motif (Assunção et al., 2013). This motif would act as a Zn-sensor, modulating bZIP19 and bZIP23 activity (e.g., affecting dimerization properties, DNA binding activity or protein-protein interactions) according to cellular Zn status (Assunção et al., 2013). Our results are in line with this hypothesis, and overall they indicate that the Zn-dependent activity of bZIP19 and bZIP23 are not controlled at the transcriptional level, but instead cellular Zn status modulates their activity at the protein level. This modulation does not seem to involve subcellular targeting, but cellular Zn sufficiency limits or represses their activity and, conversely, Zn deficiency is required for target gene expression.

### Further Insight on bZIP19 and bZIP23 Regulatory Activity

The expression of *ZIP1, ZIP4, ZIP5, NAS2*, and *NAS4* in the *bzip19/23-*OE19 and *bzip19/23*-OE23 lines reinforces their role as target genes of bZIP19 and bZIP23. They are likely playing a role in Zn transport and Zn NA-mediated distribution (Guerinot, 2000; Clemens et al., 2013) in response to Zn deficiency, and they all contain *ZDRE*-motifs, to which bZIP19 and bZIP23 bind to, in their promoter regions (Assunção et al., 2010). The regulation of *NAS* genes seems to be complex and controlled by multiple mechanisms, comprising direct or indirect regulation by FIT (FER-Like Iron Deficiency-Induced Transcription Factor), the key regulator in the Arabidopsis Fe deficiency response (Bauer et al., 2007; Palmer et al., 2013; Chen et al., 2018), being suggested to balance the interrelationship of Fe and Zn homeostasis (Chen et al., 2018). We observed that the position of *NAS2* and *NAS4* promoter *ZDRE*-motif is at the 5′UTR, comprising the ATG start codon, which might be difficult to integrate with a transcriptional activation function of bZIP19 and bZIP23, adding complexity to their regulation mechanism.

The complementation of the *bzip19/23* mutant shows that bZIP19 and bZIP23 can form functional homodimers and suggests redundancy between them, as previously reported (Assunção et al., 2010). However, analyses of single mutants, i.e., *bzip19* and *bzip23*, showed a phenotype in *bzip19* suggesting partial redundancy instead (Assunção et al., 2010; Inaba et al., 2015). Detailed analysis on these single mutants suggested that, in addition to a common set of *ZIP* target genes, bZIP19 and bZIP23 could also regulate distinct genes, being *ZIP9, ZIP4, ZIP5* predominantly regulated by bZIP19, and *ZIP12* mainly regulated by bZIP23 (Inaba et al., 2015). Our results indicated nonetheless a similar performance between *bzip19/23*-OE19 and *bzip19/23*-OE23 lines, where their target genes expression analysis and tissue elemental profiling revealed an overall comparable pattern between the two lines. This supports that bZIP19 and bZIP23 transcription factors are, to a large extent, functionally redundant. Possibly, an overlapping or a distinct regulatory activity between bZIP19 and bZIP23 might instead rely, at least partially, on their tissue- and cellular-specific expression patterns. In line with this, our results showed that there is differential tissue-specific expression, with *bZIP19* more expressed in roots and *bZIP23* more expressed in shoots. Perhaps a more relevant role for bZIP19 in the Zn deficiency response in roots could explain the reported *bzip19* Zn deficiency phenotype (Assunção et al., 2010; Inaba et al., 2015). Further studies are necessary to verify the *bZIP19* and *bZIP23* specific expression patterns under different developmental stages and growth conditions.

The tissue elemental analysis of the different lines showed, as expected, a drastic decrease in Zn tissue content at Zn deficiency. Interestingly, the difference in shoot Zn concentration between the *bzip19/23* mutant and *bzip19/23*-OE24 lines, exhibiting a severe Zn deficiency phenotype, and the wild-type, *bzip19/23*-OE19 and *bzip19/23*-OE23 lines, not exhibiting a visible Zn deficiency phenotype, is well in line with the reported critical deficiency level below 15–20 μg g^-1^ DW in leaves (Marschner, 1995). The element concentration profiling in shoots and roots did not differ strongly between all the lines at control conditions, but at Zn deficiency it provided interesting additional insight, pointing at possible interactions between the Zn deficiency regulatory network and other micronutrients homeostasis. In particular, results showed that the concentration of Mn in shoots significantly increased under Zn deficiency in wild-type, as previously observed (Campos et al., 2017), and also in the *bzip19/23*-OE19 and *bzip19/23*-OE23 lines, while there was no variation in the *bzip19/23* mutant between control and Zn deficiency. The *ZIP1* gene, target of bZIP19 and bZIP23, encodes the ZIP1 Zn transporter which is implicated in the transport of Mn, possibly in the remobilization from the vacuole (Grotz et al., 1998; Milner et al., 2013). Considering that ZIP family members are able to mediate the transport of a range of micronutrient cations, including Zn, Fe, Mn, Cu (Guerinot, 2000; Wintz et al., 2003; Eide, 2006), our results strongly suggest that bZIP19/23 targets could be involved in Mn uptake and transport, at least under Zn deficiency. Moreover, results showed a decrease in the concentration of Cu in wild-type shoots at Zn deficiency, as previously reported (Campos et al., 2017), also observed in *bzip19/23*-OE19, and to a lesser extent, *bzip19/23*-OE23 lines, but not in the *bzip19/23* mutant, suggesting crosstalk between the bZIP19/23 regulatory network and Cu homeostasis, which deserves further dissection.

In the well-documented interaction between Zn and inorganic phosphorus (Pi) homeostasis, Zn-deficient plants overaccumulate Pi in the shoot due to increased Pi uptake and/or distribution (Bouain et al., 2014). In line with this, our tissue P analysis showed, at Zn deficiency, an increase in shoot P concentration in the wild-type, *bzip19/23*-OE19 and *bzip19/23*-OE3 lines, accompanied by a decrease in root P concentration. Recently, bZIP23 was shown to be involved as a repressor in a novel pathway regulating Pi acquisition under Zn deficiency, through binding to a *ZDRE*-motif variant to which bZIP19 cannot bind (Kisko et al., 2018). This new *ZDRE*-motif is located at the 5′UTR of *LPCAT1* gene, which plays a key role in coordinating Pi homeostasis, suggesting that the binding of bZIP23 might physically block gene transcription under Zn deficiency (Kisko et al., 2018). Our results revealed no variation in shoot P content in the *bzip19/23* mutant between control and Zn deficiency, supporting the involvement of bZIP23 in this regulatory pathway. However, we did not find significant differences between the *bzip19/23*-OE19 and *bzip19/23*–OE23 lines in shoot and root P content suggesting that bZIP19 can perform the same regulatory activity as bZIP23.

### bZIP24 Does Not Play a Central Role in the Zn Deficiency Response

In addition to bZIP19 and bZIP23, the Arabidopsis bZIP F subfamily contains bZIP24, with the three members sharing a high degree of amino acid sequence similarity (Jakoby et al., 2002). Phylogenetic analysis of F-bZIP members across land plants suggests a monophyletic origin prior to seed plant evolution, and branching out of two clades: Group 1 and Group 2 (Castro et al., 2017). The bZIP19 and bZIP23, likely two paralogs resulting from a duplication event, are included in Group 1, whereas bZIP24 is in Group 2 and appears to have undergone diversifying selection (Castro et al., 2017). bZIP24 is suggested to be a negative regulator in salt stress induced acclimation responses, involved in regulating the maintenance of osmotic and ionic balance (Yang et al., 2009). We included bZIP24 in our experiments to examine any functional relation to the bZIP19 and bZIP23 Zn deficiency response. Our results showed that bZIP24 was unable to fully complement the *bzip19/*23 mutant, which is consistent with the Zn deficiency hypersensitive phenotype of *bzip19/*23 despite the endogenous bZIP24. Previously, bZIP24 was shown to form homodimers, and to localize in the nucleus and cytosol in roots of Arabidopsis bZIP24-GFP overexpressor line, with the signal in the nucleus increasing upon salt treatment (Yang et al., 2009). Our results revealed that, in addition to no effect of Zn status at the transcription level, the subcellular localization analysis in roots of the *bzip19/23-*OE24 line showed bZIP24 in the nucleus with no alteration upon Zn treatment.

Both *bzip19/23* mutant and *bzip19/23-*OE24 lines suffered from severe Zn deficiency in the hydroponically grown plants. The expression profiles of *ZIP* and *NAS* family members clearly showed that the *bzip19/23-*OE24 line had overall a pattern of expression comparable with the *bzip19/23* mutant. The tissue elemental profiling was also generally comparable between the two lines. A slightly less severe Zn deficiency phenotype was observed in the rosette of the *bzip19/23-*OE24 plants, whose seedlings showed a better performance than the *bzip19/23* mutant. It can be hypothesized that these observations rely on a modest improvement of Zn uptake and an improved distribution within the plant by NA (Clemens et al., 2013), since *bzip19/23-*OE24 in relation to *bzip19/23* mutant showed a small expression increase of *ZIP4, ZIP5* and *NAS4* genes, while the Zn concentration, at least in the shoot, of these two lines remained comparable at Zn deficiency. Nonetheless, with respect to the Zn deficiency response, our results showed that the Group 2 bZIP24 does not play a central role in the Zn deficiency response, contrary to the Group 1 bZIP19 and bZIP23, although a contribution to Zn homeostasis cannot be ruled out.

## Conclusion

We used complementation lines as a tool to further our insight on the Zn sensing and regulatory mechanism of bZIP19 and bZIP23 transcription factors. Our findings indicate that the Zn-dependent activities of bZIP19 and bZIP23 are not controlled at the transcriptional level, but at the protein level where cellular Zn status modulates their activity. This does not seem to involve interference with their nuclear localization, where cellular Zn deficiency is required for target gene expression and Zn sufficiency limits or represses their activity. We provided evidence that the two transcription factors are, to a large extent, functionally redundant, and that distinct regulatory activities may rely on their differential tissue-specific expression patterns. Our results with complementation lines also unveiled interesting indications of interactions with other micronutrient homeostasis networks, which deserve further research. Finally, we showed that the bZIP24 is not a key player in the Zn deficiency response, as bZIP19 and bZIP23 are. These Group 1 F-bZIP orthologs are possibly functionally conserved across land plants (Assunção et al., 2010; Castro et al., 2017). Considering that Zn deficiency in agricultural soils, crops and human diet represents a global challenge^2^ furthering knowledge on these regulatory mechanisms is of fundamental and practical importance.

## Author Contributions

GHL, PHC, and AGLA designed the research, performed experimental work, and analyzed the data. AC performed confocal microscopy and data analysis. AGLA drafted the manuscript revised by all authors.

## Conflict of Interest Statement

The authors declare that the research was conducted in the absence of any commercial or financial relationships that could be construed as a potential conflict of interest.

## Footnotes

1 www.ncbi.nlm.nih.gov/tools/primer-blast

2 www.harvestplus.org
