# Supplementary material for: The Arabidopsis bZIP19 and bZIP23 Activity Requires Zinc Deficiency – Insight on Regulation From Complementation Lines
Source: Front Plant Sci. 2019 Jan 22;9:1955. doi: 10.3389/fpls.2018.01955 (PMC6349776; doi:10.3389/fpls.2018.01955)
Supplement: Supplementary file 1 [file Table_1.docx]

**The Arabidopsis bZIP19 and bZIP23 activity requires zinc deficiency – insight on regulation from complementation lines**

**Authors**

Grmay H. Lilay, Pedro Humberto Castro, Ana Campilho, Ana G. L. Assunção

**Supplementary Material**

**Supplementary Figure S1, S2, S3, S4**

**Supplementary Table S1, S2, S3**

**Supplementary Methods**

**Supplementary Figures**


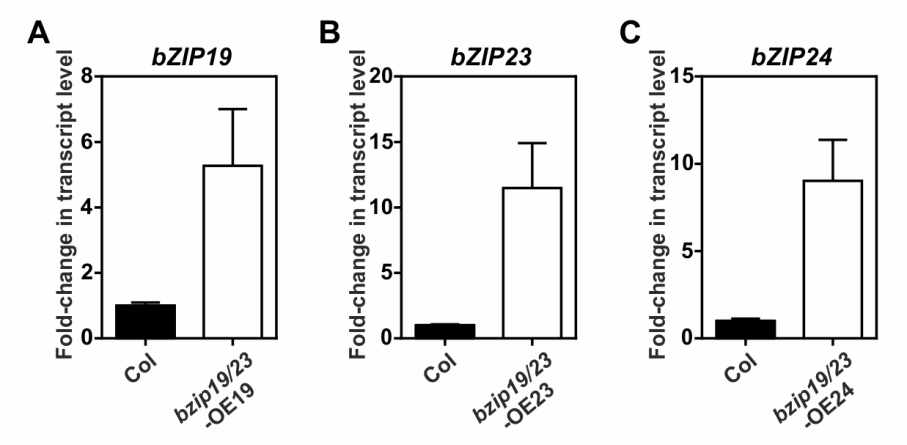


**Figure S1**. Transcript level profiles of Arabidopsis *bZIP19* (**A**), *bZIP23* (**B**) and *bZIP24* (**C**) using real-time quantitative RT-PCR, in 14-day-old seedlings of wild-type (Col) and *bzip19/23*-OE19, *bzip19/23*-OE23 or *bzip19/23*-OE24 lines, respectively, grown in MS medium. Bars represent mean fold-change in transcript level of three biological replica ±SE.


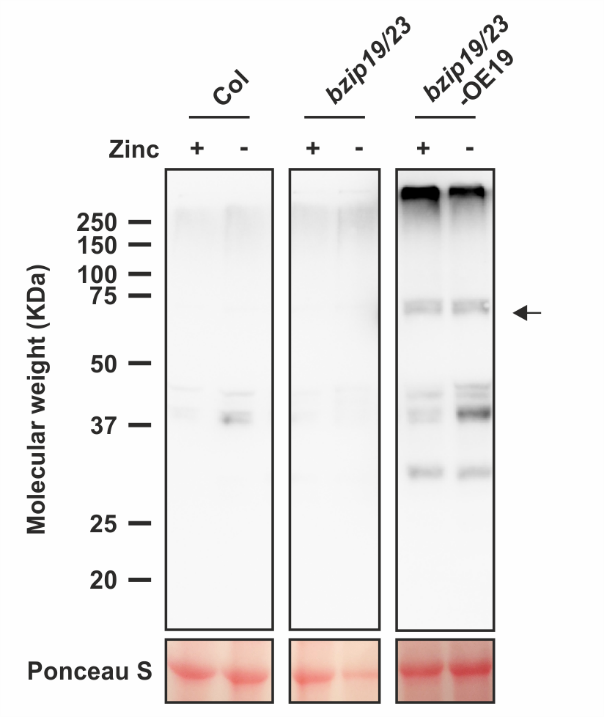


**Figure S2**. Western blot analysis of bZIP19-CFP-HA protein expressed in 10-day-old seedlings of Arabidopsis wild-type (Col), *bzip19/23* double mutant and *bzip19/23*-OE19 lines grown in control (+) or Zn-deficient (-) MS medium. Total protein per sample (20 µg) was verified with Ponceau S staining, and anti-HA primary antibody was used. Expected band for bZIP19-CFP-HA (59.17 kDa) is indicated with an arrow.


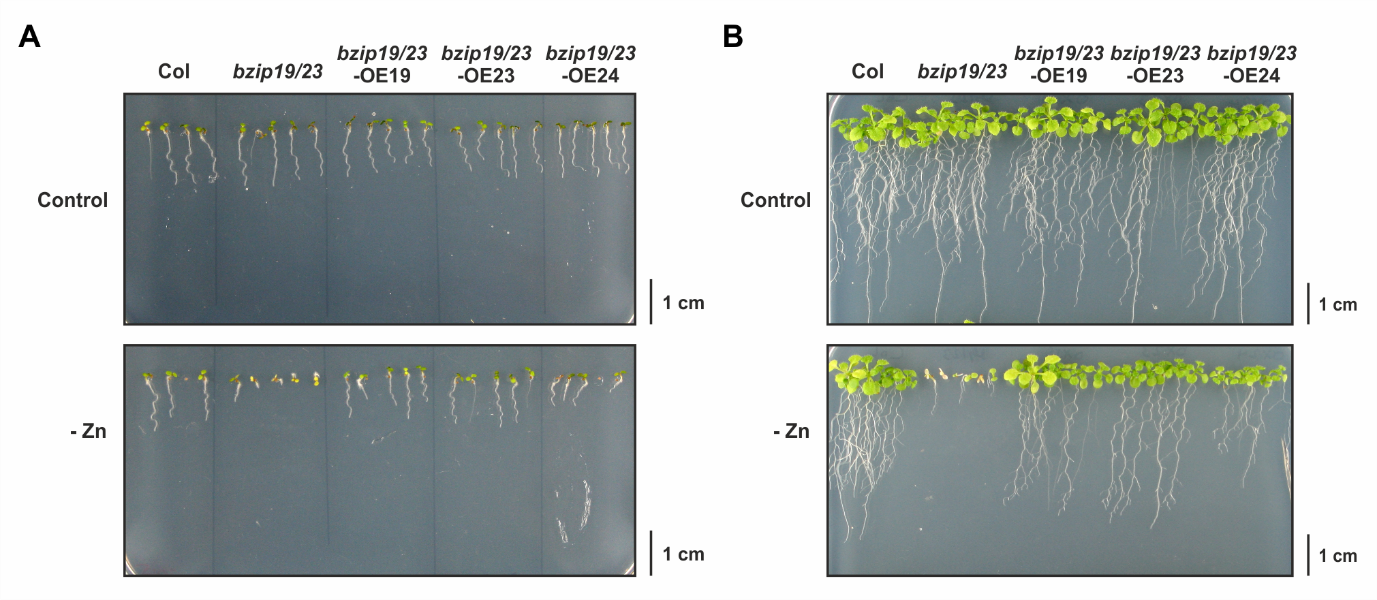


**Figure S3**. Seedlings of 5-day-old (**A**) and 14-day-old (**B**) Arabidopsis wild-type (Col), *bzip19/23* double mutant, *bzip19/23*-OE19, *bzip19/23*-OE23 and *bzip19/23*-OE24 lines, grown in control (control) or Zn-deficient (-Zn) MS medium.


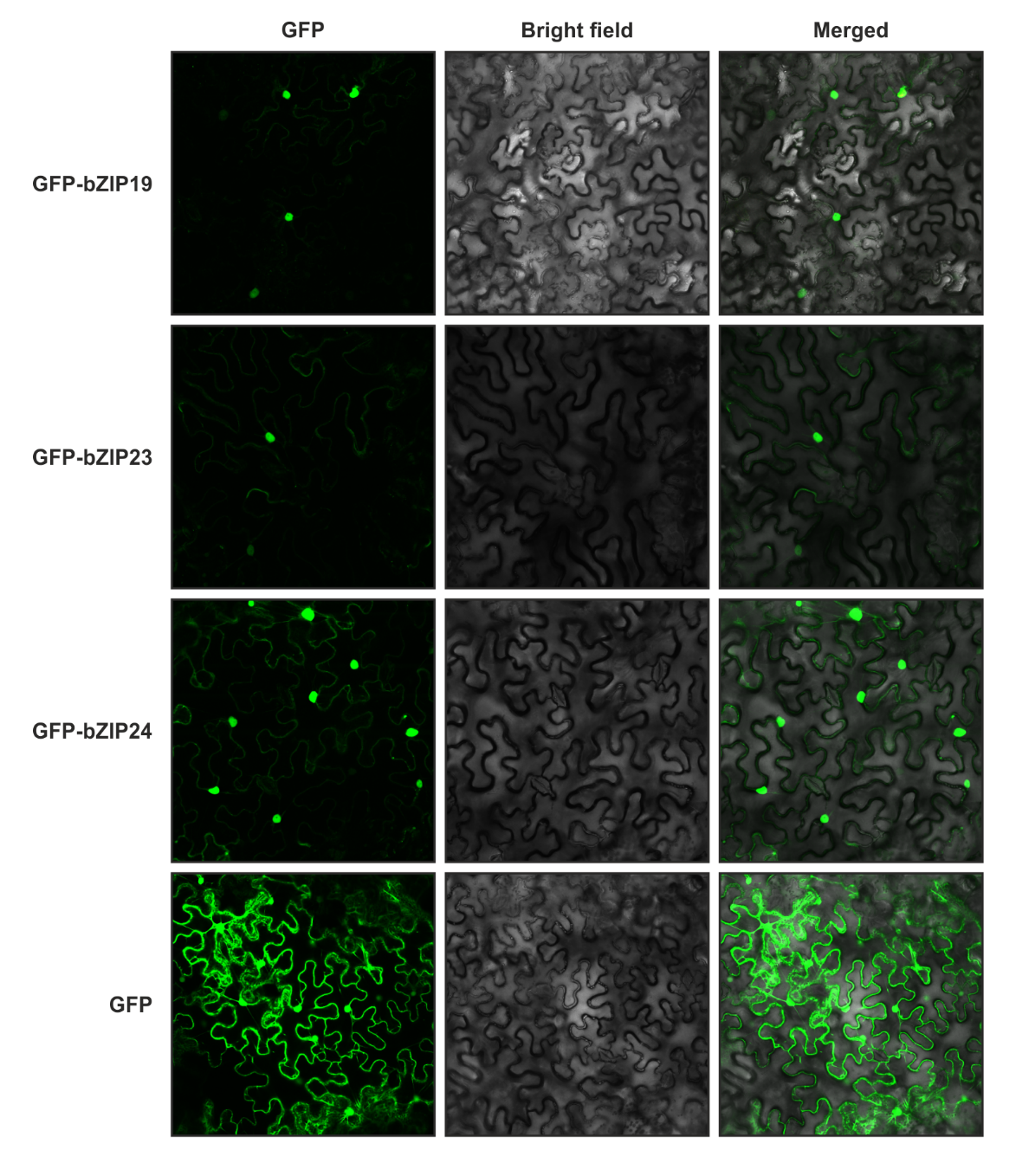


**Figure S4**. Subcellular localization analysis of GFP-bZIP19, GFP-bZIP23 and GFP-bZIP24 fusion proteins transiently expressed in *Nicotiana benthamiana* leaves. Emissions of GFP and bright-field (transmission) were visualized with confocal laser scanning microscopy (CLSM) for GFP-bZIP19, GFP-bZIP23, GFP-bZIP24 and GFP.

**Supplementary Tables**

**Table S1**. Forward (F) and reverse (R) primers, containing *Not*I (F) and *Asc*I (R) restriction enzyme sites (bold), used for cloning the CDS of Arabidopsis *bZIP19*, *bZIP23* and *bZIP24* and the promoters of *bZIP19* and *bZIP23*.

| **Primer name** | **Sequence 5’-3’** |
| --- | --- |
| bZIP19 F | GG**GCGGCCGC**CATGGAAGACGGTGAGCTTGATTTC |
| bZIP19 R | AT**GGCGCGCC**CAACTGCTCTTGATGCACGATGCCC |
| bZIP23 F | GG**GCGGCCGC**CATGGACGACGGTGAGCTTGAG |
| bZIP23 R | AT**GGCGCGCC**CAACTGCTTTCGCTGCTCGAGGCTC |
| bZIP24 F | TT**GCGGCCGC**CATGTTTTGTTGTTGCAAGGATTGC |
| bZIP24 R | GT**GGCGCGCC**CACGTGAGAATGGTGGTGGTTGAGG |
| pbZIP19 F | CG**GCGGCCGC**CCTAAAAACCTACTTTAGATCTAGG |
| pbZIP19 R | TA**GGCGCGCC**CTACTAAAACCCACCAACCACT |
| pbZIP23 F | GG**GCGGCCGC**CTGATCTTACGTGTTGAAATTTA |
| pbZIP23 R | AT**GGCGCGCC**CTACTCAAAACCCAGTAACAAAGAAG |

**Table S2**. Forward (F) and reverse (R) primers used in real-time quantitative RT-PCR analysis to determine transcript levels of Arabidopsis *bZIP19*, *bZIP23*, *bZIP24*, *ZIP1*, *ZIP4*, *ZIP5*, *NAS2*, *NAS4* and the reference gene *ACT2*.

| **Primer name** | **Sequence 5’-3’** |
| --- | --- |
| bZIP19 F | GGCTACCTTGGAAGCTGAGG |
| bZIP19 R | TGATCGAGGCACCTTCTTGG |
| bZIP23 F | CATCGATGAATGAGCAAGGGC |
| bZIP23 R | TGCTCGAGGCTCACCTTTTC |
| bZIP24 F | TCTGGTAGAGATGCAGGGGA |
| bZIP24 R | CTCTTGCCGCTTCACACATCA |
| ACT2 F | CTAAGCTCTCAAGATCAAAGGCTTA |
| ACT2 R | ACTAAAACGCAAAACGAAAGCGGTT |
| ZIP1 F | GGACACACACATGGTTCGAC |
| ZIP1 R | GATAGTGCAGCCATGAGTGG |
| ZIP4 F | GATCTTCGTCGATGTTCTTTGG |
| ZIP4 R | TGAGAGGTATGGCTACACCAGCAGC |
| ZIP5 F | CGGGATTGTTGGCGTGGAAT |
| ZIP5 R | CCAAGACCCTCGAAGCATTG |
| NAS2 F | CGACGTGGTTAATTCGGTGG |
| NAS2 R | CGCGTGGACCTTAGAGCAAT |
| NAS4 F | GTCGTTCTTGCCTCTTCCCA |
| NAS4 R | GAGCAGCCAAGAACACAACG |

**Table S3**. Tissue elemental profiling of potassium (K), calcium (Ca), magnesium (Mg) and sulphur (S) macronutrients, determined with inductively coupled plasma-mass spectrometry (ICP-MS) in 8-week-old plants of Arabidopsis wild-type (Col), *bzip19/23* double mutant, *bzip19/23*-OE19, *bzip19/23*-OE23 and *bzip19/23*-OE24 lines, grown in hydroponics with control nutrient solution. Data are represented in µg g^-1^ dry weight (DW), as means ±SE (n=4). Different letters indicate significant differences (*p* < 0.05) after one-way ANOVA followed by Tukey’s post-hoc test.

|  | **K** |  | **Ca** |  | **Mg** |  | **S** |  |
| --- | --- | --- | --- | --- | --- | --- | --- | --- |
| **Shoot** |  |  |  |  |  |  |  |  |
| Col | 45916.7 ± 786.0 | a | 55647.7 ± 516.3 | a | 7600.2 ± 76.6 | a | 7533.3 ± 165.8 | a |
| *bzip19/23* | 48311.7 ± 985.8 | ab | 55410.5 ± 751.1 | a | 7401.5 ± 108.6 | a | 7391.6 ± 173.8 | a |
| *bzip19/23*-OE19 | 50242.5 ± 82.4 | b | 57338.1 ± 725.7 | a | 7628.7 ± 201.8 | a | 8349.9 ± 194.5 | a |
| *bzip19/23*-OE23 | 49976.2 ± 434.1 | b | 57166.7 ± 440.1 | a | 7687.9 ± 77.3 | a | 7760.2 ± 181.2 | a |
| *bzip19/23*-OE24 | 48365.1 ± 1236.6 | ab | 55906.2 ± 256.6 | a | 7768.5 ± 102.4 | a | 7966.2 ± 348.6 | a |
| **Root** |  |  |  |  |  |  |  |  |
| Col | 81742.7 ± 2107.5 | a | 6526.2 ± 94.9 | a | 2222.8 ± 32.7 | a | 15549.0 ± 189.8 | a |
| *bzip19/23* | 92042.0 ± 1303.9 | ac | 6781.5 ± 83.2 | a | 2128.9 ± 32.1 | ab | 17471.0 ± 205.4 | bc |
| *bzip19/23*-OE19 | 86968.3 ± 3252.4 | ab | 6414.6 ± 146.5 | a | 2020.9 ± 32.0 | b | 16764.5 ± 348.3 | ab |
| *bzip19/23*-OE23 | 95314.0 ± 2128.4 | bc | 6709.7 ± 207.0 | a | 2171.5 ± 68.3 | ab | 18139.8 ± 250.2 | c |
| *bzip19/23*-OE24 | 99966.0 ± 1072.7 | c | 6595.1 ± 176.6 | a | 2073.1 ± 37.9 | ab | 18584.6 ± 475.2 | c |

**Supplementary Methods**

**Protein extraction and immunoblotting**

Ten-day-old seedlings of wild-type and *bzip19/23*-OE19 lines grown in control or –Zn MS medium were grinded in a microtube in liquid nitrogen, with the help of polypropylene pestles. Protein extracts were obtained by adding extraction buffer (50 mM Tris; 150 mM NaCl; 0.2 % (v/v) Triton X-100) supplemented with Complete Protease Inhibitor Cocktail (Roche), as manufacturer instructions. Following incubation for 1 h at 4°C with agitation, microtubes were centrifuged for 2 × 30 min at 16,000 *g*. The supernatant was recovered and stored at −80°C. Protein was spectrophotometrically quantified using Bradford Reagent (Sigma-Aldrich). Total protein (20 µg per sample) was separated electrophoretically in a Mini-PROTEAN TGX Precast gel using Mini-PORTEAN Cell (Bio-Rad) apparatus. Proteins were transferred to Trans-Blot Turbo Midi PVDF Transfer Packs membranes using Trans-Blot Turbo Transfer System (Bio-Rad) following standard protocol. PVDF membranes were blocked overnight at 4ºC in blocking solution (5% dry milk powder in PBST). The primary antibody Anti-HA (ab9110, Abcam) was added in a 1:4000 dilution and incubated for 2 h. Membranes were washed three times, at constant rotation for 10 min, with approximately 10 mL of PBST. The membranes were incubated for 1 h with the secondary antibody Anti-Rabbit (IgG-peroxidase, A6154, Sigma) in a 1:10,000 dilution in blocking solution. The membrane was washed as previously described and developed using the Immun-Star WesternC Kit (Bio-Rad) and a Chemidoc Touch Imaging System (Bio-Rad) for image acquisition. As protein equal loading reference, membranes were incubated for 15 min with Ponceau S solution to stain total protein.
